# 

**Published:** 1837

**Authors:** 


### BIBLIOGRAPHICAL RECORD;

### OR, QUARTERLY LIST OF NEW PUBLICATIONS IN MEDICINE AND SURGERY, AND THEIR ASSOCIATE SCIENCES.*

### American.

1. The Medical Student, or Aids to the Study of Medicine; including a Glossary of the Terms of the Science, and of the Mode of Prescribing, Bibliographical Notices of Medical Works, the regulation of different Medical Colleges of the Union, &c. &c. By Robert Dunglison, M. D., Professor of the Institutes of Medicine and Medical Jurisprudence in Jefferson Medical College. 8vo. Philadelphia, 1837.

2. Clinical Guide, for the use of Pupils engaged in the Study of Clinical Medicine, in preparing for Examination. By W. W. Gerhard, M. D., one of the Physicians of the Philadelphia Hospital, and Lecturer on Clinical Medicine. 8vo. pp. 40. Philadelphia: 1837.

3. Reports and other Documents relating to the State Lunatic Asylum at Worcester, Massachusetts. Printed by order of the Senate. 8vo. pp. 200. Boston: 1837.

4. Elements of Surgery. By Robert Liston, F. R. C. S. London and Edinburgh, Lecturer on Surgery, &c. 8vo. pp. 540. Philadelphia: 1837.

5. Animal Magnetism. Report of Dr. Franklin and others. Translated from the French, with an historical outline of the "Science," &c. 8vo. pp. 58. Philadelphia: 1837.

6. Letter to Dr. Brigham on Animal Magnetism; being an account of a remarkable interview between the author and Miss Loraina Brackett, while in a state of Somnambulism. By William L. Stone. 8vo. pp. 66. New-York: 1837.

7. Thoughts on Schools of Medicine, their Means of Instruction, and Modes of Administration, with reference to the Schools of Louisville and Lexington. By Charles Caldwell, M. D. 8vo. pp. 32. Louisville: 1837.

8. Observations on the Operation of Lithotomy; illustrated by cases from the Practice of Prof. Dudley. By J. M. Bush, M. D. 8vo. pp. 21. Lexington: 1837.

9. Statistics of the Deaf and Dumb in the State of New-York, the United States, and in various countries of Europe. By T. Romyne Beck, M. D., &c. 8vo. Albany: 1837.

10. A Discourse on some of the Diseases of the Knee-joint, delivered before the Massachusetts Medical Society, at their annual meeting, May 31, 1837. By Geo. Hayward, M. D., Professor of the Principles of Surgery and of Clinical Surgery, in Harvard University, and Surgeon to the Massachusetts General Hospital. 8vo. Boston: 1837.

11. A Treatise on Insanity, and other Disorders affecting the Mind.

*We would respectfully suggest to the authors of new works, the propriety of forwarding us copies, as soon after their publication as practicable. By so doing, they will always secure for them an early notice in our record. As things are now conducted, many of the eastern books never reach us until they are six or twelve months old.

By J. C. Pritchard, M. D., F. R. S. &c. &c. 8vo. pp. 339. Philadelphia: 1837.

BRITISH.

1. The Philosophy of Living. By Herbert Mayo, F. R. S. 8vo. London: 1837.

2. A Practical Treatise on Diseases of the Skin. By Samuel Plumbe, M. D., &c. 4th edition, with additional Engravings. 8vo. pp. 607. London: 1837.

*This work, one of the most excellent on the diseases of the skin, in the English language, has been recently republished in "Bell's Select Medical Library."*

3. A Dictionary of Practical Medicine. By J. Copland, M. D., F. R. S. Part IV. London: 1837.

4. On Deformities of the Chest and Spine. Illustrated by Plates. By Wm. Coulson, M. R. C. S., &c. Second edition, greatly enlarged. Small 4to. London: 1837.

5. A Practical Compendium of Diseases of the Skin. By Jonathan Green, M. D. 2d edition, with plates. 8vo. pp. 371. London: 1837.

6. Homæopathy briefly Examined, in a Letter to Sir H. Halford, M. D. By a Licentiate. 8vo. pp. 66. London: 1837.

7. Historical and Medical Researches into the Origin, Nature and Treatment of Syphilis. By M. Devergie, Sen. Translated from the French, by John S. Jones, Surgeon. 12mo. Edinburgh: 1837.

8. A Treatise on the Diagnosis and Treatment of Diseases of the Chest. Part I. Diseases of the Lungs and Windpipe. By William Stokes, M. D., Physician to the Meath-Hospital. 8vo. pp. 557. Dublin: 1837.

9. The Works of John Hunter, F. B. C. With Notes. Edited by James F. Palmer. Senior Surgeon of the St. George's and the St. James' Dispensary, &c. In four octavo volumes; illustrated by a volume of quarto Plates. London: 1837.

10. Guy's Hospital Reports. No. IV. April, 1837. Edited by Dr. Barlow and Dr. Babington. 8vo. pp. 310. London.

11. The Medico-Chirurgical Formulary; or a Conspectus of the Best Prescriptions. By Michael Ryan, M. D., M. R. C. 12mo. London: 1837.

12. The Philosophy of Human Nature, in its Physical, Intellectual and Moral Relations. By H. McCormac, M. D. 8vo. pp. 564. London: 1837.

13. A Treatise on the Diseases and Injuries of the Larynx and Trachea. By Frederick Ryland, Surgeon, Birmingham. 8vo. pp. 338, with Plates. London: 1837.

14. Rudiments of Physiology. By Dr. Fletcher. Part III.—on Life, as manifested in Sensation and Thought. Edited by Robert Lewins, M. D., with a Biographical Memoir of the Author. 8vo. Edinburgh: 1837.

15. The Principles of the Theory and Practice of Medicine, including a third edition of the author's work on Diagnosis. By Marshall Hall, M. D., F. R. S. 8vo. pp. 503. London: 1837.

16. A Clinical Treatise on the Endemic Fevers of the West Indies; intended as a Guide for the Young Practitioner in those Countries. By W. J. Evans, Esqr., M. R. C. S. 8vo. pp. 309. London: 1837.

17. The Principles of Surgery. By James Syme, F. R. S. E., Professor of Clinical Surgery in the University of Edinburgh. 8vo. pp. 460. Edinburgh: 1837.

*This is decidedly the best book for the student of surgery with which we are acquainted. It is the only one in the English language that is strictly constructed on the principles of the anatomy of the tissues.*

18. A Treatise on Diet; with a view to establish, on practical grounds, a System of Rules for the Prevention and Cure of the Diseases incident to a Disordered State of the Digestive Functions. By J. A. Paris, M. D., F. R. S., &c. 8vo. pp. 414. London: 1837.

19. On the Nature and Treatment of Dropsies; in four parts. By Jonathan Osborne, M. D., Fellow and late President of the King and Queen's College of Physicians, in Ireland, &c. &c. 2d edition. 8vo. pp. 134. London: 1837.

20. First Principles of Surgery; being an outline of Inflammation and its Effects. By George T. Morgan, A. M., Lecturer on Surgery in Aberdeen. 8vo. London: 1837.

21. Transactions of the Provincial Medical and Surgical Association; Vol. V. 8vo. pp. 538. London: 1837.

22. Essay on the Mineral Waters of Carlsbad, for Physicians and Patients. By Chevalier John De Carro, M. D., with Observations on the Microscopic Animalcules about the Hot Springs of Carlsbad. By Mr. A. J. C. Corda, of Prague; and a Flora of Carlsbad, by Professor C. B. Prest, of Prague. 12mo. pp. 136.

23. Practical Observations on the Venereal Disease, and on the use of Mercury. By Abraham Colles, M. D., one of the Surgeons of Steevens's Hospital, and lately Professor of Surgery in the Royal College of Surgeon's, in Ireland. 8vo. pp. 351. London: 1837.

### French.

1. Cours De Pathologie Interne, Professé a la Facultie De Medicine De Paris. Par M. G. Andral, M. D., Professeur a Ladite Facultie, Membre De L'Academie Royale De Medicine, &c. &c. Recucilli et Redigi. Par Amédée Latour, M. D. In three vols. 8vo. Paris: 1837.

2. Maladies Des Organes de la Voix. Premier Memoire—-Sur Laryngite Aigue. Par Bureaud Riofrey, M. D. 8vo. Paris: 1837.

3. Clinique Médicale de l'Hôpital de la Charité; ou Exposition Statistique des Diverses Maladies traitees a la Clinique de cet Hôpital. Par J. Bouillaud, M. D., Professeur De Clinique, &c. In two volumes, octavo, pp. 508–488. Paris: 1837.

4. Maladies des Jeunes Filles pendant d'Epoque de l'accroissement. Premier Memoire—De la Chlorose. Par Bureaud Riofrey, M. D. Octavo, pp. 34. Paris: 1836.

5. Recherches Sur L'Affection Tuberculeuse des os. Par A. Nelaton, M. D.; Interne des Hôspitaux, &c. 8vo. pp. 70. Paris: 1837. *To be noticed in our next number.*

6. Precis D' Anatomie Comparée; ou Tableau De l'Organisation considerée Dans l'Ensemble de la Serie Animale. Par H. Hollard, M. D., Professeur D'Anatomie et Physiologie Comparée, &c. 8vo. pp. 586. Paris: 1837. *An excellent work, which we shall probably present, ere long, in an English dress.*

7. Traité Pratique de la Phthisie Laryngée, Avec Planches. Par A. Trousseau et H. Belloc, M. D. 8vo. pp. 488. Paris: 1837.

8. Manuel de Medicine Operatoire. Par J. F. Malgaigne, M. D., Professeur, &c., Ed. d. pp. 773. Paris: 1837.

GERMAN.

1. Encyclopädisches Worterbuch der Medicinischen Wissenschaften. Herausgegeben von den Professoren der Medicinischen Facultât zu Berlin—D. W. Busch, C. F. V. Grafe, C. W. Hufeland, H. F. Lunk, J. Müller, Band xiv. (Geburtmutter-Gift.) 8vo. S. 752. Berlin: 1836.

2. Chirurgische Kupfertafeln. Von Dr. Robert Froriep. Heft 69–71. 4to. Weimer: 1837.

3. De Vitâ et Opinionibus Theophrasti Paracelsi—-Dissertatio. Auctore A. F. Bremer. 8vo. pp. 191. Hanniæ: 1836.

4. Zur Praxis der Geburtshûlfe. Beobachtungen und Bemarkungen dur der Academischen Entbindungs anstalt zu Göttingen Während der beiden Jahre 1822 and 1832. Von Dr. J. Osiander, Professor der Medicin zu Göttingen. 8vo. pp. 143. Hannover: 1837.

5. Denkwürdigkeiten in der Artzlichen Praxis. Von Dr. J. H. Kopp, Kurfûstlich Geheimen Ober Medicinalrathe, &c. Zu Hanau. Dritter Band. 8vo: s. 407. Frankfurt, a. m. 1836.

6. Encyclopädie der Gesammten Medicinischen and Chirurgischen Praxis, mit einschluss der Geburtshülfe, der Augenheilkunde und der Operativchirurgie. In verein mit mehreren praktischen arzten und Wundärzten. Herausgegeben von George T. Most, D. M., P. & C., u. s. w. Zweite Auflage. Heft 4, 5, 6, 7, 8, 9. (Dysphobia-Phthisis.) 8vo. Leipzig: 1836-37.

7. De Aneurismatibus Arteriarum Cerebri. Auctore A. A. A. Stumpff. Quarto; pp. 36. Berolini: 1836.

8. Darstellungen und Ausichten zur Vergleichung der Medicin in Frankreich, England, und Deutschland. Von Dr. Adolph Mühry. 8vo. s. 238. Hannover: 1836.

